# Giant J waves in a critically ill patient with COVID‐19 pneumonia

**DOI:** 10.1002/hsr2.1253

**Published:** 2023-05-10

**Authors:** Hai Zou

**Affiliations:** ^1^ Department of Critical Care Fudan University Shanghai Cancer Center Shanghai China; ^2^ Department of Oncology, Shanghai Medical College Fudan University Shanghai China

**Keywords:** COVID‐19, critically ill, ECG, giant J waves

A 85‐year‐old male patient was admitted to the hospital due to fever and cough for 7 days. The patient had a hypertension history. Medication for hypertension was not clear. He did not have a personal or family history of heart disease, arrhythmia or any other conditions. He was confirmed with coronavirus disease 2019 (COVID‐19) by positive severe acute respiratory syndrome coronavirus 2 nucleic acid test and a viral pneumonia presentation in computed tomography (CT) scan. The patient presented with acute respiratory illness and CT (Figure 1A) combined with CXR (Figure 2) that shows diffuse infiltrates bilaterally suggestive of the acute respiratory distress syndrome and was transferred to intensive care unit for mechanical ventilation and further management on February. 20. 2020. The core temperature was 37°C. Mean arterial pressure (MAP) was 78 mmHg without vasoactive medications. Electrolyte disturbance or acute kidney injury was not found on admission. A 12‐lead electrocardiogram (ECG) examination was performed right after admission (Figure 1B). With SIMV mode (FiO_2_ 0.6−0.8, rate 18, PS 12, PEEP 8 mmH_2_O, Insp. time 1.3 s) and repeated prone position, Hyoxemia worsened 4 days later. The patient was sedated with midazolam and remifentanil. His Ramsay score was 2. CT scan indicated progression of pneumonia on February 25, 2020 (Figure 1C). Ultrasonic cardiogram reported no organic or functional changes (LVEF 60%) on February 26, 2020 while NT‐proBNP rose from 1201 to 8528 pg/mL. Estimated glomerular filtration rate dropped from 79.4 to 44.2 mL/min/1.73 m^2^ (calculated by CKD‐EPI equation). Lactic acid rose from 5.4 to 9.8 mmol/l. MAP dropped to 68 mmHg. These findings indicated multiple organ dysfunction syndrome due to irreversible hypoxiaNorepinephrine and sodium bicarbonate injection was prescript to correct shock and acidosis. Magnesium polarization liquid and trimetazidine was applied for better myocardial metabolism and cell membrane stabilization. conservative fluid management was replaced by fluid resuscitation with a 500−1000 mL fluid balance per day. A sharp decline of oxygen saturation and blood pressure was found February 28, 2020 as soon as ECG monitoring showed a significant sinus bradycardia with J wave and ST‐T segment abnormality, confirmed by ECG test (Figure 1D). The acquired giant J seemed to indicated severe myocardial ischemia, which might be related with hypoxemia or acidosis and possibly led to cardiac arrest. So sodium bicarbonate injection was ordered and invasive arterial blood gas analysis (ABG) was tested immediately. ABG revealed severe respiratory failure and respiratory acidosis(pH 7.096, pO2 42 mmHg, pCO2 58.2 mmHg, HCO_3_‐act, r 17.5 mmol/L, HCO_3_‐ stad,r 14.4 mmol/L, K^+^ 4.8  mmol/L, Ca^2+^ 1.0  mmol/L, Lac 11.8  mmol/L). Calcium gluconate, glucose and insulin was used for hyperkalemia and hypocalcemia. Volume expansion and norepinephrine was used to reverse circulatory collapse. However, cardiac arrest took place in 3 h after the ECG test despite medication and cardio‐pulmonary rescue.

**Figure 1:**
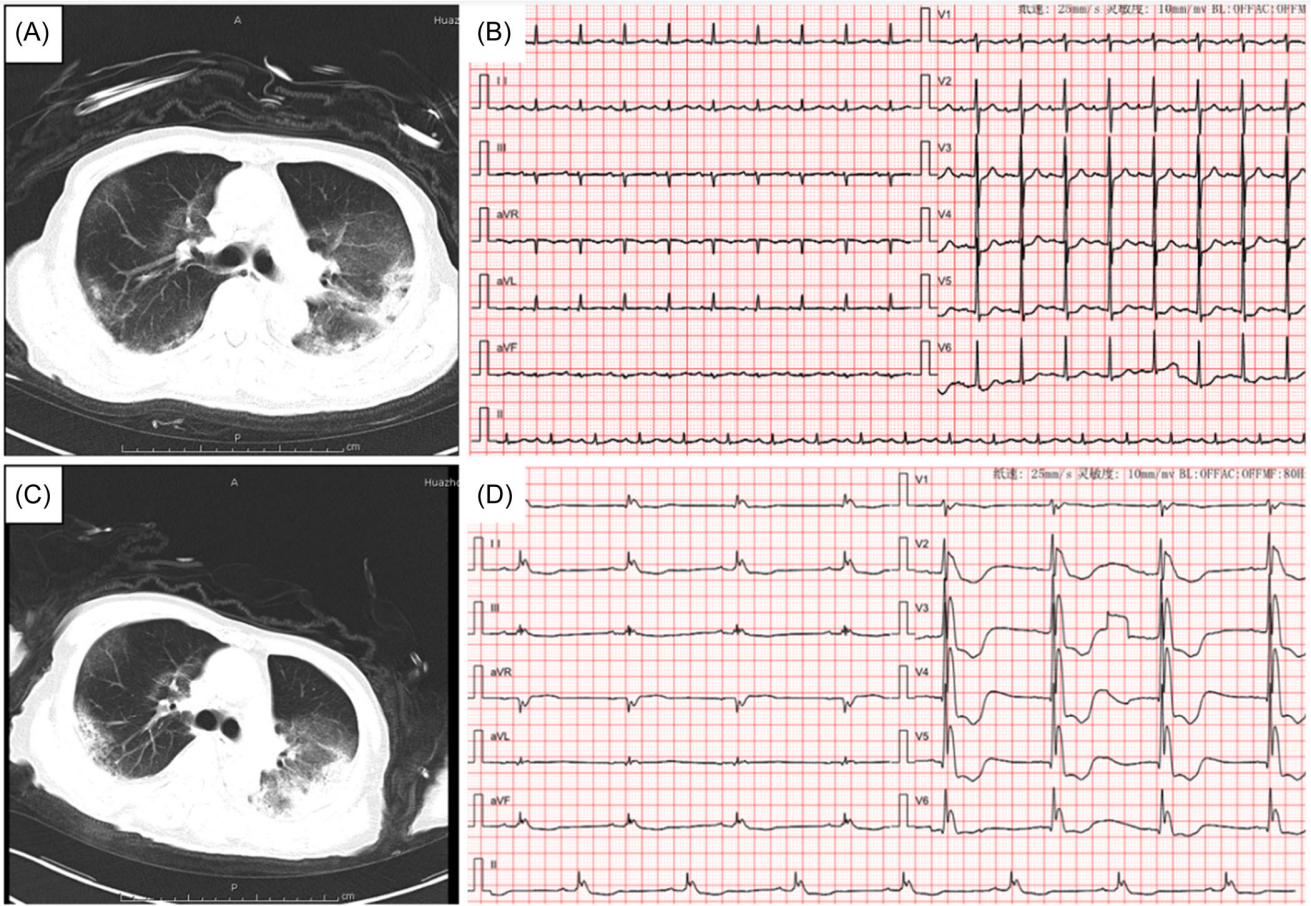
ECG report and CT of critically ill patient with COVID‐19 pneumonia. CT, computed tomography; ECG, electrocardiogram.

**Figure 2:**
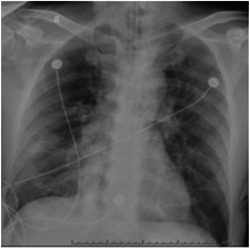
Chest X‐ray of critically ill patient with COVID‐19 pneumonia.

## CONFLICT OF INTEREST STATEMENT

The authors declare no conflict of interest.

## TRANSPARENCY STATEMENT

The lead author Hai Zou affirms that this manuscript is an honest, accurate, and transparent account of the study being reported; that no important aspects of the study have been omitted; and that any discrepancies from the study as planned (and, if relevant, registered) have been explained.

## ETHICS STATEMENT

As this report is a “clinical image,” no formal ethics committee vote is available. This research was carried out in accordance with recognized standards (the Declaration of Helsinki, as revised in 2013). Informed consent was obtained from the patient for publication of the clinical image and related accompanying images.

## Data Availability

Data sharing not applicable to this article as no data sets were generated or analyzed.

